# Global Trends in Ischemic Heart Disease-Related Mortality From 2000 to 2019

**DOI:** 10.1016/j.jacadv.2025.101904

**Published:** 2025-06-19

**Authors:** Vikash Jaiswal, Jef Van den Eynde, Yusra Mashkoor, Helen Huang, Vamsi Garimella, Sulochana Khadka, Tushar Kumar, Akash Jaiswal, Wilbert Aronow, Maciej Banach, Gregg C. Fonarow

**Affiliations:** a Department of Cardiovascular Research, Larkin Community Hospital, South Miami, Florida, USA; b The Blalock Taussig Thomas Pediatric and Congenital Heart Center, Department of Pediatrics, Johns Hopkins School of Medicine, Johns Hopkins University, Baltimore, Maryland, USA; c Department of Cardiovascular Sciences, KU Leuven & Congenital and Structural Cardiology, UZ Leuven, Leuven, Belgium; d Department of Internal Medicine, Dow University of Health Sciences, Karachi, Pakistan; e Royal College of Surgeons in Ireland, University of Medicine and Health Sciences, Dublin, Ireland; f Department of Internal Medicine, University of Miami, Miami, Florida, USA; g Department of Internal Medicine, UPMC, Harrisburg, Pennsylvania, USA; h Department of Cardiothoracic and Abdominal Radiology, University of Washington, Seattle, Washington, USA; i Department of Geriatric Medicine, All India Institute of Medical Science, New Delhi, India; j Department of Cardiology, Westchester Medical Center, Valhalla, New York, USA; k Department of Preventive Cardiology and Lipidology, Medical University of Lodz (MUL), Lodz, Poland; l Faculty of Medicine, The John Paul II Catholic University of Lublin, Lublin, Poland; m Department of Cardiology and Adult Congenital Heart Diseases, Polish Mother’s Memorial Hospital Research Institute (PMMHRI), Lodz, Poland; n Ciccarone Center for the Prevention of Cardiovascular Disease, Johns Hopkins University School of Medicine, Baltimore, Maryland, USA; o Liverpool Centre for Cardiovascular Science at University of Liverpool, Liverpool John Moores University and Liverpool Heart & Chest Hospital, Liverpool, United Kingdom; p Ahmanson-UCLA Cardiomyopathy Center, Ronald Reagan UCLA Medical Center, Los Angeles, California, USA

**Keywords:** cardiovascular pathology, global trends, ischemic heart disease, mortality

## Abstract

**Background:**

Ischemic heart disease (IHD) remains one of the leading causes of morbidity and mortality across the globe, and disparities exist based on sex and geographic region.

**Objectives:**

This study investigates global trends in IHD mortality and examines disparities based on sex and geographic regions.

**Methods:**

IHD mortality data from 105 countries were obtained from the World Health Organization Mortality Database. Crude mortality rates (CMRs) and age-standardized mortality rates (ASMRs) per 100,000 individuals were calculated, with average annual percentage change (AAPC) analyzed using joinpoint regression. Regional and sex-specific trends were assessed using stratified analyses of CMR and ASMR.

**Results:**

Globally, CMR declined from 138 per 100,000 (95% CI: 131-145) in 2000 to 106 per 100,000 (95% CI: 102-114) in 2019 (AAPC: −1.79, 95% CI: −1.93 to −1.66). Similarly, ASMR declined from 104 per 100,000 (95% CI: 99-108) to 65.5 (95% CI: 62-69) in 2019 per 100,000 (AAPC: −2.16, 95% CI: −2.13 to −2.20). Regionally, CMRs decreased in Oceania, Europe, and North America, while they rose in Asia, Africa, and Central and South America. ASMRs declined worldwide except in Africa (AAPC: 1.33, 95% CI: 1.30-1.36). Males showed higher mortality than females, but both sexes demonstrated decreasing trends, with males having a steeper decline. In age groups across all regions, Africa showed an upward trend, while other regions demonstrated declines.

**Conclusions:**

While global IHD mortality has declined from 2000 to 2019, disparities by geographic region and sex persist. Implementing targeted health awareness programs and collaborative global health efforts are crucial for addressing these inequalities.

Ischemic heart disease (IHD) remains one of the leading causes of morbidity and mortality across the globe. According to a recent study published in *JACC* by Mensah et al,^1^ based on Global Burden of Disease 2022 data, there were approximately 315 million prevalent cases of IHD worldwide, resulting in 9.24 million deaths. In recent years, there have been a shift in the global trends in IHD-related mortality due to various factors, including demographic changes, lifestyle, and advancements in the health care system. However, disparities exist among high-income countries, which were able to bring down mortality and morbidity with better health care management and knowledge, but these gains were lagging in low- and middle-income countries, especially with higher prevalent risk factors such as obesity, diabetes, hypertension, and smoking.^2^^,^^3^ Understanding worldwide trends in IHD-related mortality is essential for guiding recommendations by cardiology experts, national health authorities, and the World Health Organization (WHO). These insights are crucial for addressing disparities and mitigating the rising mortality risk in low- and middle-income countries, which often face challenges due to limited health care infrastructure or delayed adoption of medical advancements. Hence, with this study, we aim to evaluate the global trends in mortality from IHD and determine the sex- and age-based estimates.

## Methods

### Data source

This analysis utilized data from the WHO mortality database, which compiles official national statistics provided directly by government authorities in each participating country.^4^ Mortality records in the database indicate the underlying cause of death, defined as the primary disease leading to death, based on the International Classification of Diseases (ICD). The WHO database is a widely recognized source for epidemiological studies on various diseases, including both communicable and noncommunicable conditions.^5^^,^^6^ For this study, we analyzed trends in IHD mortality from 2000 to 2019, identifying IHD-related deaths using ICD codes (ICD-9 codes 410-414 and ICD-10 codes I20-25). Population age distribution data for each country at midyear were sourced from the United Nations World Population Prospects 2022.^7^ Institutional review board approval was not required for this study, as it relied exclusively on publicly available government data, containing no individually identifiable information.

### Data analysis

Crude mortality rates (CMRs) and age-standardized mortality rates (ASMRs) per 100,000 people were calculated for deaths related to IHD. CMRs were obtained by dividing the number of IHD deaths by the total population in a group of countries each year. ASMRs were calculated by dividing the number of deaths in each age category (≤39, 40-64, 65-74, 75-84, and ≥85 years) by the population in that age group. ASMR for each country group, including a 95% CI, was estimated using the WHO World Standard Population and formulas developed by Tiwari et al.^8^^,^^9^

To assess trends in CMR and ASMR across world regions, we used the “segmented” package in R Statistical Software, which models data as consecutive linear segments on a log scale, joined at specific points (joinpoints) where the segments converge.^10^ Comparative analyses evaluated regional differences in CMR and ASMR from 2000 to 2019. Furthermore, analyses based on sex and age were conducted to examine mortality trends separately for males and females, as well as across various age groups, over the study period. The average annual percent change (AAPC) and corresponding 95% CIs were calculated, with slopes considered significantly increasing or decreasing if they statistically differed from zero. The significance level was set at *P* = 0.05. All analyses were conducted using R Statistical Software (version 4.1.1).

## Results

### Characteristics of regions and populations

Mortality data from the WHO database included 105 countries across 6 regions (Europe, Asia, North America, Africa, Central and South America, and Oceania) between 2000 and 2019 were included in this study. The median annual total population for all 6 regions during the observation period was 2.3 billion, with a median of 18.8 million deaths from any cause per year (Table 1). Europe had the highest population count (0.7 billion), life expectancy at birth (77.4 years), and crude death rate (10.4 deaths per thousand), while the highest annual birth count was observed in Central and South America (9.6 million per year).

**Table 1:** Population Characteristics and Mortality Data Across Regions (2000-2019)

	Total	Africa	Asia	Central and South America	Europe	North America and the Caribbean	Oceania
Population, in millions	2,253.0 (2,171.5, 2,401.2)	139.2 (129.0, 151.5)	470.1 (452.8, 571.0)	523.2 (493.6, 538.2)	722.2 (715.7, 727.1)	372.6 (355.8, 385.9)	25.7 (24.6, 27.5)
Median age, y	30.0 (27.2, 35.0)	27.0 (25.9, 28.7)	29.0 (28.1, 31.1)	25.0 (23.7, 26.4)	38.5 (37.1, 40.1)	32.0 (30.1, 33.9)	32.39 (31.1, 36.3)
Births, in thousands	33,392.6 (31,796.1, 34,935.4)	3,592.0 (3,215.3, 3,848.7)	7,683.2 (6,829.4, 8,187.0)	9,579.5 (9,407.3, 9,773.5)	7,382.5 (7,267.3, 7,909.3)	4,800.9 (4,756.5, 4,846.8)	354.4 (320.3, 370.0)
Deaths from any cause, in thousands	18,754.8 (17,870.1, 19,812.82)	1,120.9 (1,108.5, 1,153.8)	3,362.8 (2,976.6, 3,911.4)	3,080.9 (2,873.7, 3,274.9)	8,056.8 (7,849.1, 8,155.7)	2,961.7 (2,896.3, 3,135.6)	171.7 (165.9, 181.4)
Crude death rate from any cause, per thousands	7.10 (5.96, 7.72)	7.5 (7.13, 7.69)	5.8 (5.71, 5.86)	5.9 (5.89, 5.98)	10.40 (10.34, 10.56)	7.5 (7.26, 7.84)	6.98 (6.67, 7.09)
Life expectancy at birth, y	75.0 (73.0, 76.7)	71.0 (69.0, 71.9)	75.0 (74.3, 77.0)	73.0 (72.4, 74.5)	77.4 (76.2, 78.8)	76.0 (74.9, 76.2)	76.51 (75.7, 82.1)

Values are median (IQR).

### Crude and age-standardized mortality rates stratified by region

**Figure 1:**
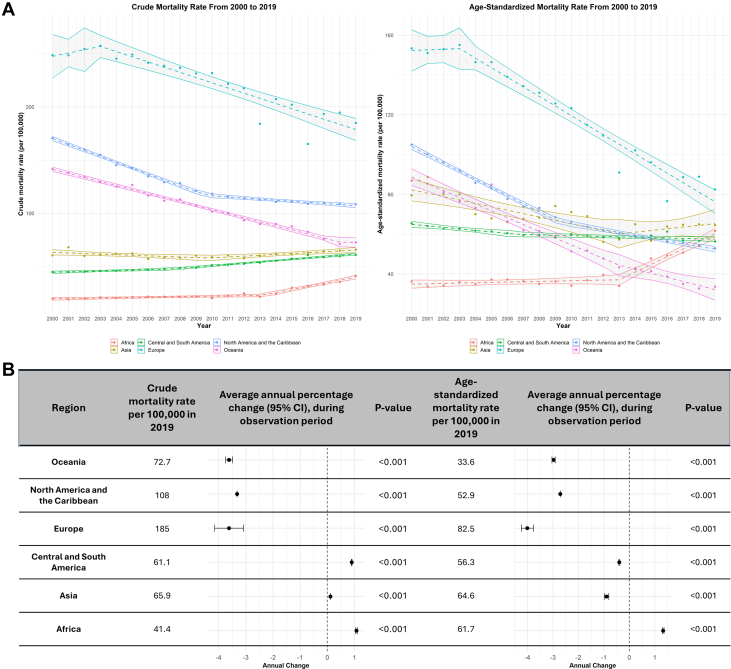
Mortality Trends for Ischemic Heart Disease per 100,000 (2000-2019), Stratified by Region (A) Annual trends in CMR and ASMR for IHD per 100,000 (2000-2019), stratified by region. (B) IHD mortality rates per 100,000 in 2019 and average annual percent change (2000-2019), stratified by region. ASMR = age-standardized mortality rate; CMR = crude mortality rate; IHD = ischemic heart disease.

The total number of IHD-related deaths decreased from 2,901,461 in 2000 to 2,498,637 in 2019. Globally, CMR declined from 138 per 100,000 (95% CI: 131-145) in 2000 to 106 per 100,000 (95% CI: 102-114) in 2019, with an AAPC of −1.79 (95% CI: −1.93 to −1.66). Regionally, CMRs decreased in Oceania, Europe, and North America, while they rose in Asia, Africa, and Central and South America (Figure 1A). The CMR per 100,000 in 2019 was highest in Europe at 108, followed by North America and Oceania (Figure 1B). However, all 3 regions exhibited negative AAPCs that were statistically significant. The AAPC for Europe and Oceania are both −3.63 (Europe, 95% CI: −3.09 to 4.15; Oceania, 95% CI: −3.75 to −3.49). In North America, the AAPC was −3.32 (95% CI: −3.32 to −3.32). On the other hand, Africa experienced a positive AAPC of 1.07 (95% CI: 1.04-1.10) with the lowest CMR of 41.4 per 100,000 across all regions. Central and South America exhibited an AAPC of 0.89 (95% CI: 0.89-0.91).

Similarly, ASMR also dropped from 104 per 100,000 (95% CI: 99-108) in 2000 to 65.5 per 100,000 (95% CI: 62-69) in 2019, with an AAPC of −2.16 (95% CI: −2.13 to −2.20) (Figure 1A). However, ASMRs declined worldwide except in Africa, with an ASMR of 61.7 per 100,000 (AAPC: 1.33, 95% CI: 1.30-1.36) (Figure 1B). In Europe, the ASMR was highest at 82.5 per 100,000 with an AAPC of −4.00 (95% CI: −4.24 to −3.77). This was followed by Oceania (AAPC −2.97, 95% CI: −3.04 to −2.91) and North America (AAPC −2.71, 95% CI: −2.71 to −2.71). As such, adjusting for age creates a significant shift in the mortality rate, with most countries decreasing except for Africa over time.

### Country-level trends in mortality rates

Generally, Eastern European countries exhibited high CMR and ASMR in 2000 and 2019 (Supplemental Figure 1). We identified that Ukraine was represented as the most significant outlier due to high CMR and ASMR in both years, followed by Lithuania in comparing CMR and Kazakhstan in comparing ASMR (Supplemental Figure 1). However, Eastern European countries such as Russia, Latvia, Slovakia, and Estonia exhibited improvements in CMR comparing 2000 to 2019. The same is observed for Georgia and Kyrgyzstan. In comparison, there were improvements in ASMR across Asian and African countries such as Uzbekistan, Kazakhstan, Kyrgyzstan, and Georgia.

### Crude and age-standardized mortality stratified by sex

**Figure 2:**
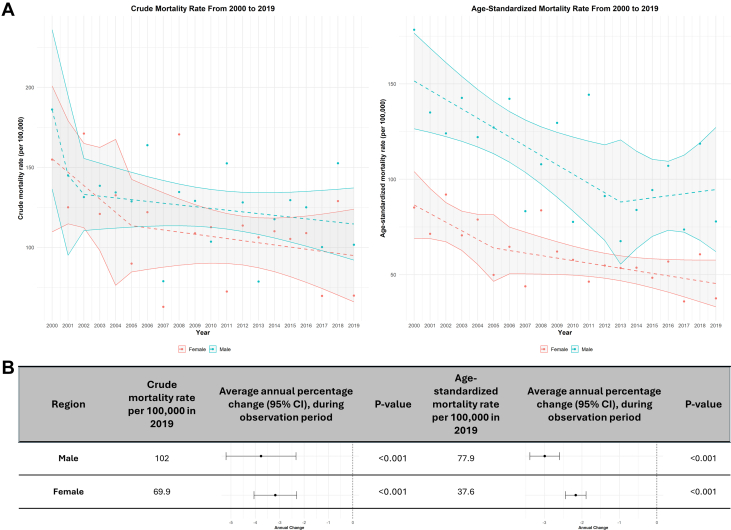
Mortality Trends for Ischemic Heart Disease per 100,000 (2000-2019), Stratified by Sex (A) Annual trends in CMR and ASMR for IHD per 100,000 (2000-2019), stratified by sex. (B) IHD mortality rates per 100,000 in 2019 and AAPC (2000-2019), stratified by sex. AAPC = average annual percentage change; ASMR = age-standardized mortality rate; CMR = crude mortality rate; IHD = ischemic heart disease.

In 2000, the total number of deaths due to IHD was 1,490,491 for males and 1,432,954 for females. By 2019, these numbers had decreased to 1,356,768 for males and 1,149,200 for females. Males exhibited higher mortality than females, but both sexes demonstrated decreasing trends, with more visible improvements in male mortality (Figure 2A). CMR in males was 102 per 100,000 people, with an AAPC of −3.77 (95% CI: −5.20 to −2.33), whereas females exhibited a CMR of 70 per 100,000 people and an AAPC of −3.18 (95% CI: −4.06 to −2.30) (Figure 2B). ASMR was lower than CMR across all the genders, with improvement in males (AAPC −2.99, 95% CI: −3.39 to −2.60) and females (AAPC −2.17, 95% CI: −2.44 to −1.89). These differences were all statistically significant.

### Crude mortality rates across different age groups within regions

**Figure 3:**
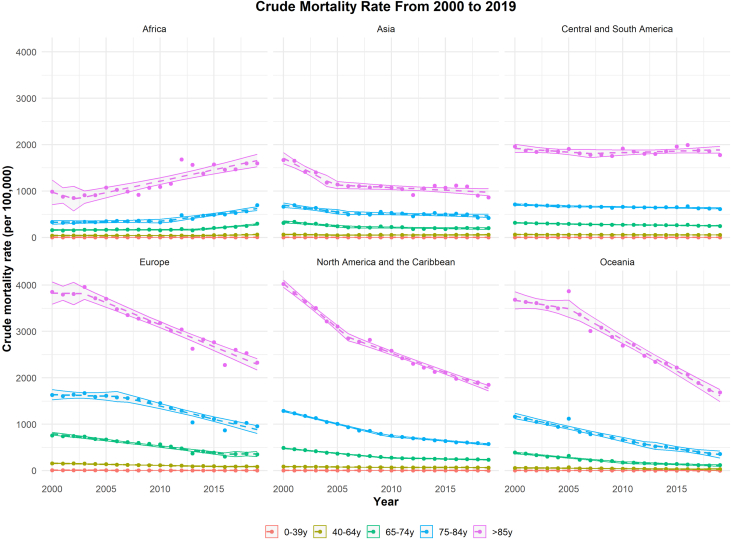
Regional Trends in Age-Specific Crude Mortality Rate per 100,000 for Ischemic Heart Disease (2000-2019)

A general trend is observed whereby annual change in regard to mortality increases based on increasing age groups, except for Central and South America, a region where trends seemed unchanged between 2000 to 2019 (Supplemental Figure 2). We also identified increasing CMR in Africa within increasing age groups, while all of the other regions were significantly decreasing (Figure 3). In Asia, the AAPC for individuals over 85 years of age was −38.5 (95% CI: −40.1 to −36.2) followed by those between 75 and 84 years of age with an AAPC of −12.1 (95% CI: −12.9 to −11.3). This is similar in Europe, where those >85 years of age have an AAPC of −80.9 (95% CI: −87.1 to −74.7). For those between 75 and 84 years old, AAPC was −36.9 (95% CI: −40.9 to −38.3), whereas the 65- to 74-year-old group exhibited an AAPC of −23.1 (95% CI: −24.56 to −21.59).

While there are similar trends in North America and the Caribbean, this region exhibited significantly high AAPC in individuals >85 years old (AAPC: −118.5, 95% CI: −119.5 to −117.7). This is similar in Oceania as well, with an AAPC of −108.12 (95% CI: −111.05 to −105.2) for individuals >85 years old and then an AAPC of −43.79 (95% CI: −44.71 to −42.86) within the 75- to 84-year-old group. This was also similar among those aged 65 to 74 years old, with an average AAPC of −13.5. However, Africa seemed to exhibit positive AAPC with reduced CMR. In individuals >85 years old, the AAPC was 36.0 (95% CI: 29.3-42.9), followed by 75 to 84 years old exhibiting an AAPC of 15.7 (95% CI: 15.5-15.8). In Central and South America, the highest AAPC was among individuals aged 65 to 74 years old, with an AAPC of −4.3 (95% CI: −4.6 to −3.9).

## Discussion

The study analyzed IHD mortality data from 105 countries (2000-2019), revealing global declines in CMR and ASMR, with notable regional differences. Oceania, Europe, and North America showed decreases, while Africa and Central and South America experienced increases. Some Eastern European countries, such as Ukraine and Lithuania, had persistently high mortality rates. Males had higher mortality than females, though both sexes showed declining trends (Central Illustration). Mortality generally increased with age, with a downward trend in most regions, except Africa, which saw an increase, and Central and South America, where rates remained stable.

**Central Illustration:**
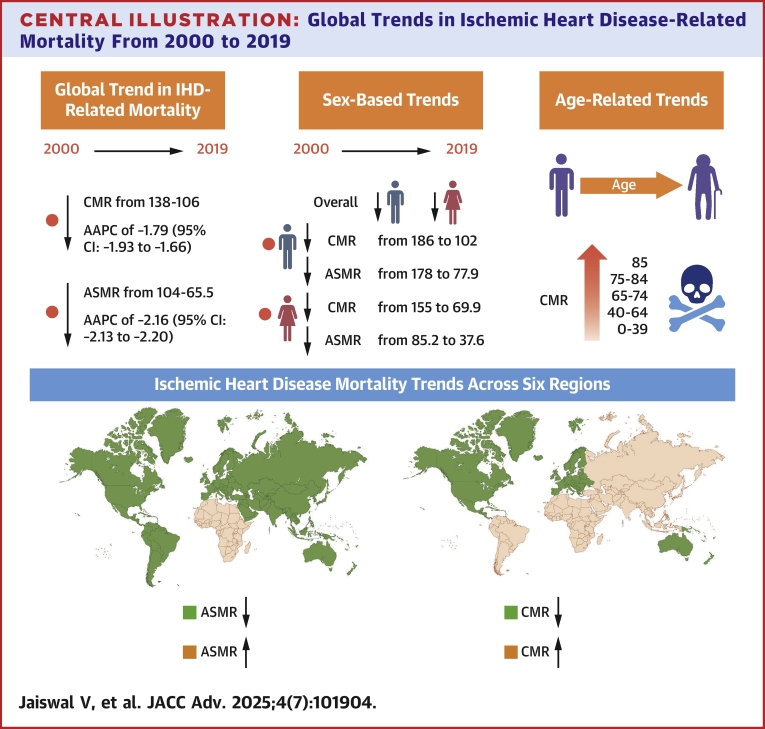
Global Trends in Ischemic Heart Disease-Related Mortality From 2000 to 2019 Abbreviations as in Figure 2.

Our study highlights a global decline in IHD mortality, as evidenced by reductions in both CMR and ASMR, signaling progress in health care, public health policies, and cardiovascular disease management.^11^ Key contributing factors likely include heightened awareness of cardiovascular risk factors, improved access to health care, and the widespread adoption of prevention strategies such as smoking cessation programs, effective blood pressure control, and the use of statins.^12^ Notably, the decrease in ASMR compared to CMR suggests that, when accounting for age demographics, the improvements in IHD mortality are even more pronounced. Though the improvements in medical technology, availability of rapid-response emergency medical services and health care systems, advanced interventional methods, and therapeutic drugs have likely enhanced survival rates, population growth and aging have led to an increase in absolute IHD cases.^13^ Thus, consistent with prior studies, our findings emphasize that while IHD mortality has decreased, it remains a significant public health challenge, requiring strategies that focus not only on reducing mortality but also on enhancing patient quality of life.^14^^,^^15^

Our study indicates that despite these global improvements, regional variations remain striking. While Oceania, Europe, and North America experienced substantial declines in IHD-related CMRs, other regions like Asia, Africa, and Central and South America saw increasing rates during the observation period, highlighting variations due to economic status, health care infrastructure, and lifestyle-related risk factors.^16^ Recent improvements in IHD mortality rates in the regions with high socio-demographic index highlight the benefits of targeted health care investments, with 3 factors contributing predominantly to this decline: policies promoting risk factor reduction, improved acute IHD treatments (such as angioplasty and thrombolysis), and better implementation of secondary prevention strategies.^17^

In contrast, some regions, such as Africa and Latin America, have seen increasing IHD mortality due to health care access issues, underfunded health care systems, and growing risk factors like hypertension, obesity, and diabetes.^18^, ^19^, ^20^, ^21^, ^22^ Furthermore, in these regions, economic poverty contributes to elevated stress levels, which, in turn, promotes the development of atherogenic factors associated with heart disease.^23^, ^24^, ^25^ A previous study also showed artificially low case-diagnosis rates in low-socio-demographic index areas due to misclassification and lack of resources, further hindering accurate assessment and intervention for IHD.^26^ Additionally, rapid urbanization and the marketing of tobacco, processed foods, and sugary drinks to low- and middle-income countries exacerbate lifestyle-related risk factors, further driving the increase in IHD mortality in regions already facing health disparities.^26^ Some of the Eastern European countries remain unique in this scenario, with persistently high IHD mortality rates despite broader declines in Europe. Factors like high smoking prevalence, poor dietary habits, and alcohol consumption likely contribute significantly to the high burden of IHD in these countries.^17^ Socioeconomic inequality and higher exposure to stress have further sustained this trend.^27^^,^^28^ Thus, our findings underscore the need for region-specific interventions, emphasizing affordable health care, expanded infrastructure, and robust prevention programs in these high-burden regions.^29^, ^30^, ^31^

Our study, in line with prior research, indicates persistently higher mortality rates in males when compared to females.^32^ Moreover, the ASMR for IHD is higher in males, while the CMR is similar between males and females. This likely occurs because females generally live longer, and as they age, they become more susceptible to developing IHD. As a result, their CMR eventually aligned with those of males.^33^ Interestingly, females tend to engage more in primary prevention strategies like healthy eating and physical activity and may respond better to certain therapies, such as angiotensin-converting enzyme inhibitors (ACEI), than males.^34^ By contrast, males are more likely to undergo secondary prevention procedures like revascularization, yet females often experience better IHD outcomes.^34^ Behavioral factors such as smoking, alcohol use, high-sodium diets, and stress are more prevalent in males, leading to poor glucose regulation and hyperglycemia, while females generally show lower alcohol consumption, better hypertension control, and higher high-density lipoprotein levels.^34^ Interestingly, sociocultural factors, such as dual employment and caregiving roles, exacerbate IHD risks, especially in females from developing countries.^35^^,^^36^

While age-standardized mortality from IHD has decreased in high-income countries since the 1980s, the aging populations in these countries contribute to a slower decline in overall mortality rates.^17^ In contrast, the rising populations and age-specific death rates in low- and middle-income countries mean that they will account for the majority of global IHD deaths moving forward.^17^ Our study also found higher mortality rates in the elderly population when compared to their younger counterparts. Aging is a major risk factor for IHD, with older individuals being more susceptible to hypertension, diabetes, dyslipidemia, and other comorbidities.^37^^,^^38^ Additionally, poor mental health and lack of social support exacerbate IHD risk in the elderly.^37^ In younger adults, improved compliance with medical treatments and lifestyle changes has led to better disease management.^39^ However, in consistency with a prior study, our study found that the IHD mortality rates for younger age groups decline at a slower pace, likely due to competing causes of death, such as dementia or heart failure, which are more common in older populations.^21^ To reduce premature IHD deaths in younger adults, efforts like smoking cessation, healthier diets, and early hypertension treatment are crucial.^39^ Additionally, as global aging accelerates, more research is needed to address the care needs of elderly patients with IHD.

### Study limitations

This study has several limitations. Although ICD codes are widely used for death certification, misclassification of IHD deaths remains unavoidable due to the complexity of cardiovascular diseases and the frequent presence of comorbidities.^39^ Variations in reporting practices between countries, particularly in cases with multiple contributing causes or limited medical documentation, may result in the over- or under-reporting of IHD deaths.^17^ Moreover, this study did not account for sociocultural or ethnic differences, which can influence IHD mortality patterns, as genetic and racial factors significantly impact disease prevalence and outcomes.^35^ Data quality and availability also vary widely across countries, with low- and middle-income nations facing challenges such as incomplete datasets, delays in reporting, and diagnostic inaccuracies.^38^^,^^39^ The analysis period was restricted to 2000 to 2019 due to the lack of complete and reliable mortality data for many countries beyond 2019 in the WHO mortality database. Furthermore, the onset of the COVID-19 pandemic in 2020 substantially altered global mortality patterns, which could have confounded IHD mortality trends.^40^^,^^41^ To ensure consistency and avoid the impact of pandemic-related anomalies, this study focused on the prepandemic period.

## Conclusions

This study shows a global decline in IHD mortality rates from 2000 to 2019. While most regions improved, Africa and Eastern Europe remain exceptions with higher rates, indicating the need for targeted interventions. Males consistently had higher mortality rates than females, with a more substantial decline observed in males. Older age groups experienced higher mortality than younger ones. The findings highlight significant progress in reducing IHD mortality, reflecting improvements in health care and public health initiatives over the 2 decades. Continued focus on prevention, early detection, and equitable treatment is needed to ensure sustained progress in combating IHD worldwide.

Perspectives **COMPETENCY IN PATIENT CARE AND PROCEDURAL SKILLS:** Epidemiologists, physicians, and policymakers should be aware of the disparities among the IHD-related mortality trends, which, although declining, show increasing trends in certain regions such as Africa and Eastern Europe.

**TRANSLATIONAL OUTLOOK:** Advancing the management of IHD through novel approaches and public awareness about the diet, exercise, and risk factors will play a critical role in reducing the mortality among IHD patients. Timely hospitalization and the best-suited interventions may lead to crucial protection. Medical infrastructure as well needs to be upgraded with all advanced facilities.

## Funding support and author disclosures

Dr Fonarow has consulted for Abbott, Amgen, AstraZeneca, Bayer, Boehinger Ingelheim, Cytokinetics, Eli Lilly, Johnson & Johnson, Medtronic, Merck, Novartis, and Pfizer. Dr Banach has received research grant(s)/support from 10.13039/100002429Amgen, Daiichi Sankyo, Mylan/Viatris, and 10.13039/100004339Sanofi and has served as a speaker and consultant for Adamed, Amgen, Daiichi Sankyo, Esperion, Exceed Pharma, Kogen, KRKA, Menarini, Mylan, Novartis, Novo Nordisk, Pfizer, Polpharma, Sanofi-Aventis, Servier, Teva, and Zentiva. All other authors have reported that they have no relationships relevant to the contents of this paper to disclose.

## Footnotes

Appendix For supplemental figures, please see the online version of this paper.

## References

[1] Mensah G.A., Fuster V., Murray C.J.L., Roth G.A., Global Burden of Cardiovascular Diseases and Risks Collaborators (2023). Global burden of cardiovascular diseases and risks, 1990-2022. J Am Coll Cardiol.

[2] Mensah G.A., Fuster V., Murray C.J.L., Roth G.A., Global Burden of Cardiovascular Diseases and Risks Collaborators (2023). Global burden of cardiovascular diseases and risks, 1990-2022. J Am Coll Cardiol.

[3] GBD 2021 Diseases and Injuries Collaborators (2024). Global incidence, prevalence, years lived with disability (YLDs), disability-adjusted life-years (DALYs), and healthy life expectancy (HALE) for 371 diseases and injuries in 204 countries and territories and 811 subnational locations, 1990-2021: a systematic analysis for the Global Burden of Disease study 2021. Lancet.

[4] World Health Organization (Accessed September 10, 2024). WHO Mortality Database.

[5] Barco S., Valerio L., Ageno W. (2021). Age-sex specific pulmonary embolism-related mortality in the USA and Canada, 2000-18: an analysis of the WHO Mortality Database and of the CDC multiple cause of death database. Lancet Respir Med.

[6] Baum P., Winter H., Eichhorn M.E. (2022). Trends in age- and sex-specific lung cancer mortality in Europe and Northern America: analysis of vital registration data from the WHO Mortality Database between 2000 and 2017. Eur J Cancer.

[7] Nationen V. (2022).

[8] Ahmad O.B., Boschi-Pinto C., Lopez A.D., Murray C.J.L., Lozano R., Inoue M. (2001). Age standardization of rates: a new WHO standard. Geneva World Heal Organ.

[9] Tiwari R.C., Clegg L.X., Zou Z. (2006). Efficient interval estimation for age-adjusted cancer rates. Stat Methods Med Res.

[10] Kim H.J., Fay M.P., Feuer E.J., Midthune D.N. (2000). Permutation tests for joinpoint regression with applications to cancer rates. Stat Med.

[11] Sattar N., Gill J.M.R., Alazawi W. (2020). Improving prevention strategies for cardiometabolic disease. Nat Med.

[12] Stolpe S., Kowall B., Stang A. (2021). Decline of coronary heart disease mortality is strongly affected by changing patterns of underlying causes of death: an analysis of mortality data from 27 countries of the WHO European region 2000 and 2013. Eur J Epidemiol.

[13] Bairey Merz C.N., Pepine C.J., Walsh M.N., Fleg J.L. (2017). Ischemia and No obstructive coronary artery disease (INOCA): developing evidence-based therapies and research agenda for the next decade. Circulation.

[14] Moran A.E., Forouzanfar M.H., Roth G.A. (2014). Temporal trends in ischemic heart disease mortality in 21 world regions, 1980 to 2010: the Global Burden of Disease 2010 study. Circulation.

[15] Moran A.E., Forouzanfar M.H., Roth G.A. (2014). The global burden of ischemic heart disease in 1990 and 2010: the Global Burden of Disease 2010 study. Circulation.

[16] Gwon J.G., Choi J., Han Y.J. (2020). Community-level socioeconomic inequality in the incidence of ischemic heart disease: a nationwide cohort study. BMC Cardiovasc Disord.

[17] Finegold J.A., Asaria P., Francis D.P. (2013). Mortality from ischaemic heart disease by country, region, and age: statistics from World Health Organisation and United Nations. Int J Cardiol.

[18] Yusuf S., Rangarajan S., Teo K. (2014). Cardiovascular risk and events in 17 low-, middle-, and high-income countries. N Engl J Med.

[19] Mokdad A.H., Forouzanfar M.H., Daoud F. (2016). Health in times of uncertainty in the eastern Mediterranean region, 1990-2013: a systematic analysis for the Global Burden of Disease study 2013. Lancet Glob Health.

[20] Lanas F., Soto A. (2022). Trends in mortality from ischemic heart disease in the region of the Americas, 2000-2019. Glob Heart.

[21] Rosengren A., Smyth A., Rangarajan S. (2019). Socioeconomic status and risk of cardiovascular disease in 20 low-income, middle-income, and high-income countries: the Prospective Urban Rural Epidemiologic (PURE) study. Lancet Glob Health.

[22] Marcus M.E., Manne-Goehler J., Theilmann M. (2022). Use of statins for the prevention of cardiovascular disease in 41 low-income and middle-income countries: a cross-sectional study of nationally representative, individual-level data. Lancet Glob Health.

[23] Rostami R., Moradinazar M., Moradi S. (2024). Impact of dietary risk on global ischemic heart disease: findings from 1990-2019. Sci Rep.

[24] Brink A.J., Aalbers J. (2009). Strategies for heart disease in Sub-Saharan Africa. Heart.

[25] Yuyun M.F., Sliwa K., Kengne A.P., Mocumbi A.O., Bukhman G. (2020). Cardiovascular diseases in Sub-Saharan Africa compared to high-income countries: an epidemiological perspective. Glob Heart.

[26] Mensah G.A., Roth G.A., Sampson U.K. (2015). Mortality from cardiovascular diseases in sub-Saharan Africa, 1990-2013: a systematic analysis of data from the Global Burden of Disease study 2013. Cardiovasc J Afr.

[27] Frost M.J., Tran J.B., Khatun F., Friberg I.K., Rodríguez D.C. (2018). What does it take to Be an effective national steward of digital health integration for health systems strengthening in low- and middle-income countries?. Glob Health Sci Pract.

[28] Ginter E. (1998). Cardiovascular disease prevention in Eastern Europe. Nutrition.

[29] Yusuf S., Joseph P., Rangarajan S. (2020). Modifiable risk factors, cardiovascular disease, and mortality in 155 722 individuals from 21 high-income, middle-income, and low-income countries (PURE): a prospective cohort study. Lancet.

[30] Danaei G., Finucane M.M., Lu Y. (2011). National, regional, and global trends in fasting plasma glucose and diabetes prevalence since 1980: systematic analysis of health examination surveys and epidemiological studies with 370 country-years and 2·7 million participants. Lancet.

[31] Biloglav Z., Medaković P., Ćurić J. (2024). Morbidity and mortality trends of ischemic heart disease and medical interventions in mediterranean countries—pre-COVID analysis: Croatia, Slovenia, France, Italy, and Spain. Appl Sci.

[32] AlBadri A., Wei J., Mehta P.K. (2017). Sex differences in coronary heart disease risk factors: rename it ischaemic heart disease!. Heart.

[33] Gao Z., Chen Z., Sun A., Deng X. (2019). Gender differences in cardiovascular disease. Med Nov Technol Devices.

[34] Shen N., Liu J., Wang Y. (2024). The global burden of ischemic heart disease attributed to high fasting plasma glucose: data from 1990 to 2019. Heliyon.

[35] Valtorta N.K., Kanaan M., Gilbody S., Ronzi S., Hanratty B. (2016). Loneliness and social isolation as risk factors for coronary heart disease and stroke: systematic review and meta-analysis of longitudinal observational studies. Heart.

[36] Mortensen J., Dich N., Lange T. (2018). Weekly hours of informal caregiving and paid work, and the risk of cardiovascular disease. Eur J Public Health.

[37] Zhang L., Tong Z., Han R. (2023). Global, regional, and national burdens of ischemic heart disease attributable to smoking from 1990 to 2019. J Am Heart Assoc.

[38] Shu T., Tang M., He B. (2024). Assessing global, regional, and national time trends and associated risk factors of the mortality in ischemic heart disease through Global Burden of Disease 2019 study: population-based study. JMIR Public Health Surveill.

[39] Wu P., Yu S., Wang J., Zou S., Yao D.S., Xiaochen Y. (2023). Global burden, trends, and inequalities of ischemic heart disease among young adults from 1990 to 2019: a population-based study. Front Cardiovasc Med.

[40] Roth G.A., Vaduganathan M., Mensah G.A. (2022). Impact of the COVID-19 pandemic on cardiovascular health in 2020: JACC State-of-the-Art Review. J Am Coll Cardiol.

[41] Makanjuola S., Shantikumar S. (2024). The impact of the COVID-19 pandemic on non-COVID-associated mortality: a descriptive longitudinal study of UK data. Public Health Pract (Oxf).

