# Supplementary material for: Global Trends in Ischemic Heart Disease-Related Mortality From 2000 to 2019
Source: JACC Adv. 2025 Jun 19;4(7):101904. doi: 10.1016/j.jacadv.2025.101904 (PMC12221645; doi:10.1016/j.jacadv.2025.101904)
Supplement: Supplementary data [file mmc1.docx]

**Supplemental Figure 1.** Country-Level Trends in IHD Mortality (2000-2019)

**
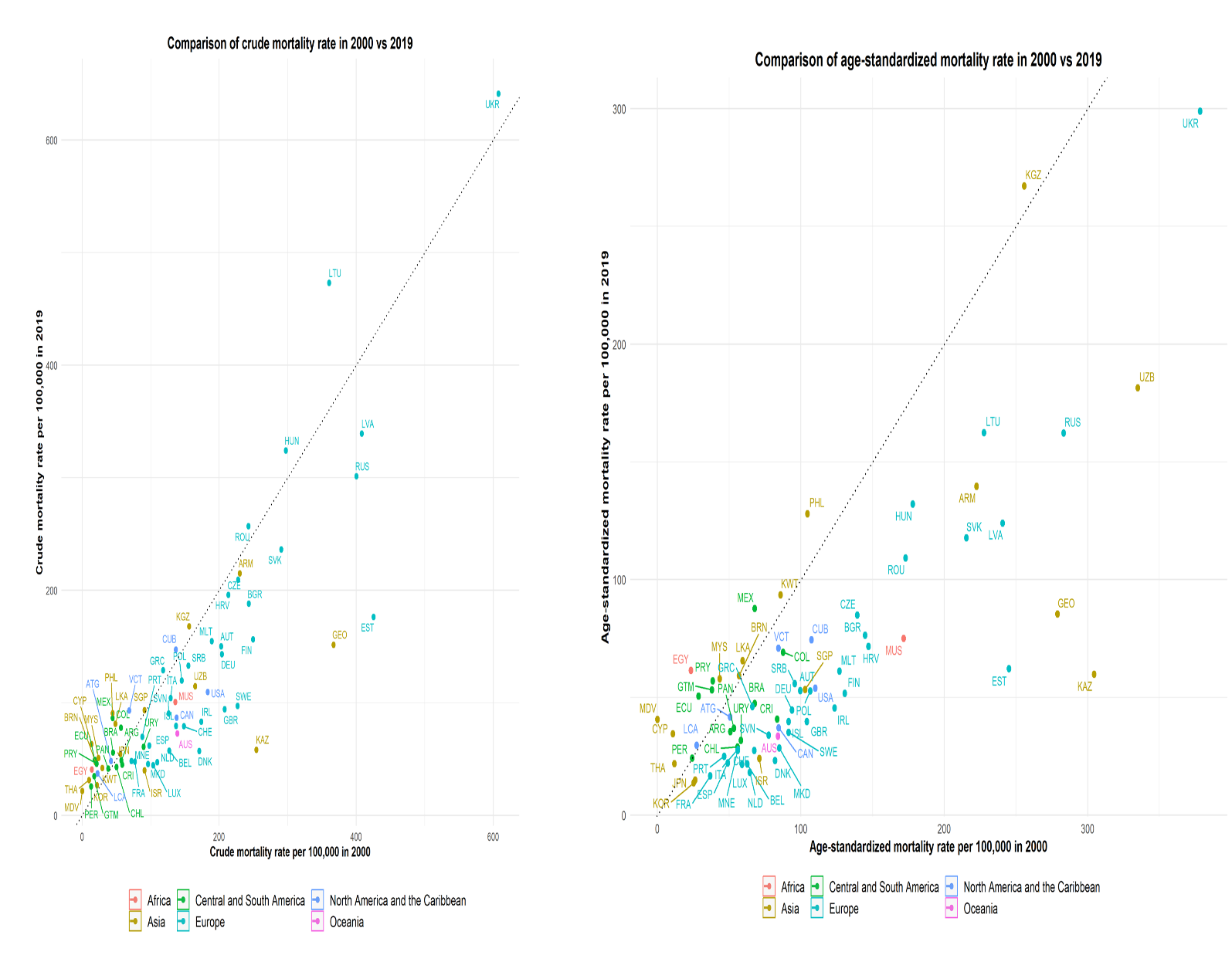
**

Algeria (DZA), Antigua and Barbuda (ATG), Armenia (ARM), Australia (AUS), Austria (AUT), Bahrain (BHR), Barbados (BRB), Belgium (BEL), Brazil (BRA), Brunei Darussalam (BRN), Bulgaria (BGR), Canada (CAN), Chile (CHL), Colombia (COL), Costa Rica (CRI), Croatia (HRV), Cyprus (CYP), Czech Republic (CZE), Denmark (DNK), Dominican Republic (DOM), Ecuador (ECU), Egypt (EGY), Estonia (EST), Finland (FIN), France (FRA), Georgia (GEO), Germany (DEU), Greece (GRC), Hungary (HUN), Iceland (ISL), Ireland (IRL), Israel (ISR), Italy (ITA), Japan (JPN), Kazakhstan (KAZ), Kyrgyzstan (KGZ), Kuwait (KWT), Latvia (LVA), Lithuania (LTU), Luxembourg (LUX), Malaysia (MYS), Maldives (MDV), Malta (MLT), Mauritius (MUS), Mexico (MEX), Montenegro (MNE), Netherlands (NLD), New Zealand (NZL), Norway (NOR), Panama (PAN), Peru (PER), Philippines (PHL), Poland (POL), Portugal (PRT), Romania (ROU), Russia (RUS), Saint Vincent and the Grenadines (VCT), Singapore (SGP), Slovakia (SVK), Slovenia (SVN), South Korea (KOR), Spain (ESP), Sri Lanka (LKA), Sweden (SWE), Switzerland (CHE), Taiwan (TWN), Thailand (THA), Tunisia (TUN), Turkey (TUR), Ukraine (UKR), United Kingdom (GBR), United States (USA), Uruguay (URY), Uzbekistan (UZB), Vietnam (VNM).

**Supplemental Figure 2.** Age-Group-Specific CMR in 2019 and AAPC for IHD Mortality Across Regions (2000-2019)

**
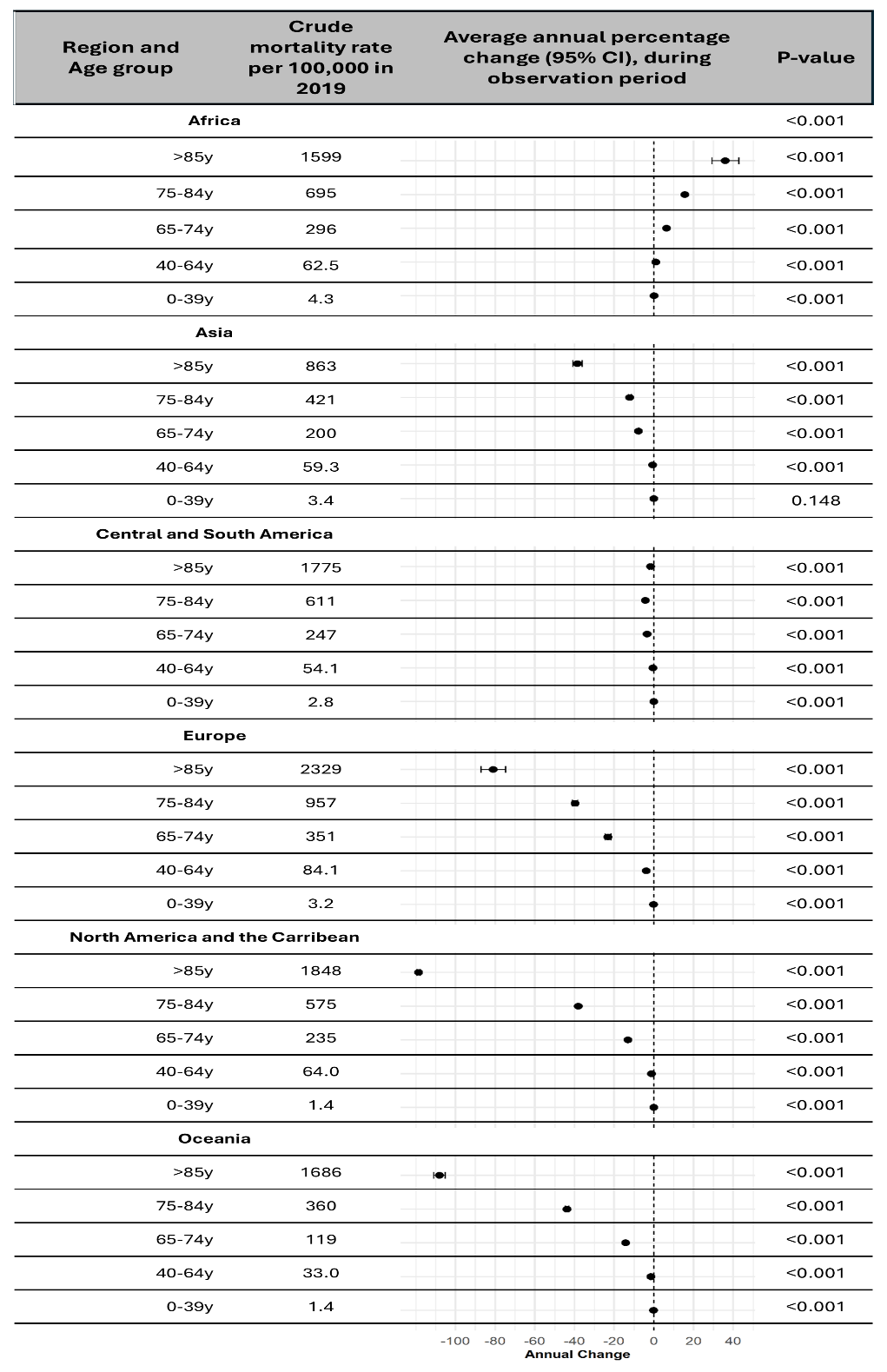
**
